# Duodenal neuroendocrine tumor after bilateral breast cancer with type 1 neurofibromatosis: a case report

**DOI:** 10.1186/s40792-024-01827-8

**Published:** 2024-01-29

**Authors:** Sho Fujiwara, Nozomi Koyamada, Koji Miyazawa, Yuriko Saiki, Akira Horii, Shukichi Miyazaki

**Affiliations:** 1 Department of Surgery, Iwate Prefectural Chubu Hospital, 17-10 Murasakino, Kitakami, , Iwate 024-8507 Japan; 2 Department of Molecular Pathology, Tohoku University School of Medicine, Sendai, Miyagi 980-8575 Japan; 3 Department of Surgery, Columbia University Irving Medical Center, 622 West 168th St, New York, NY 10032 USA; 4 Department of Surgery, Tohoku University Graduate School of Medicine, Sendai, Miyagi 980-0872 Japan; 5 Department of Investigative Pathology, Tohoku University Graduate School of Medicine, Sendai, Miyagi 980-8575 Japan; 6 Office of Medical Education, Tohoku University School of Medicine, Sendai, Miyagi 980-0872 Japan; 7 Department of Surgery, South Miyagi Medical Center, Ogawara, Shibata, Miyagi 989-1253 Japan

**Keywords:** Neuroendocrine tumor, Pancreaticoduodenectomy, Type 1 neurofibromatosis, Von Recklinghausen disease, Breast cancer, Screening

## Abstract

**Background:**

Young women with NF1 are at a high risk of developing breast cancer. Although they are at risk for abdominal tumors, such as gastrointestinal stromal tumors and neuroendocrine tumors, follow-up strategies for other tumors after breast cancer have not yet been established. Here, we present a case of duodenal neuroendocrine tumor found during follow-up after bilateral mastectomy for breast cancer with type 1 neurofibromatosis (NF1), for which pancreaticoduodenectomy (PD) and lymphadenectomy were performed.

**Case presentation:**

A 46-year-old woman with NF1 was referred to our hospital for treatment of a duodenal submucosal tumor. Her previous operative history included bilateral mastectomy for breast cancer: right total mastectomy and left partial mastectomy performed 9 and 5 years ago, respectively. Her daughter was confirmed to have NF1, but her parents were unclear. Although she had no recurrence or symptoms during the follow-up for her breast cancer, she wished to undergo 18-fluorodeoxyglucose–positron emission tomography (FDG–PET) for systemic screening. FDG–PET demonstrated FDG accumulation in the duodenal tumor with a maximum standardized uptake value of 5.78. Endoscopy revealed a 20-mm-diameter tumor in the second duodenal portion, and endoscopic biopsy suggested a NET G1. We performed PD and lymphadenectomy for complete. She was doing well without recurrence and was followed up with PET tomography–computed tomography.

**Conclusions:**

Early detection of gastrointestinal tumors is difficult, because most of them are asymptomatic. Gastrointestinal screening is important for patients with NF1, and PD with lymphadenectomy is feasible for managing duodenal neuroendocrine tumors, depending on their size.

## Background

Type 1 neurofibromatosis (NF1), also known as von Recklinghausen disease, is a type of neurofibromatosis with an autosomal dominant pattern of inheritance [1]. Patients with NF1 have a high risk of developing benign and malignant tumors, particularly breast cancer [2]. However, the risk of other tumors remains unclear. Duodenal neuroendocrine tumors are rare tumors that require complete resection [1]. However, early detection is difficult. The incidence of NF1 with symptomatic gastrointestinal tumors accounts for < 5% of cases [3]. Furthermore, the management of periampullary neuroendocrine tumor (NET) remains controversial with respect to metastasis.

Recent guidelines suggest that the risk and follow-up for gastrointestinal tumor with NF1 patients. National Comprehensive Cancer Network Guidelines version 3.2023 recommended to start the annual mammogram at 30 years and consider MRI as screening for breast cancer in NF1 patients [4]. It mentions the risk of gastrointestinal stromal tumor (GIST) as well and recommended referral to NF1 specialist for management, but they did not suggest the details. Japanese guideline does not recommend to routinely checkup for the asymptomatic gastrointestinal tumor [5]. It suggests following up every 1 year or a few years observation for adult patients with NF1 and recommends gastrointestinal screening in case of bleeding or abdominal pain [5].

Here, we present a case of duodenal neuroendocrine tumor found during follow-up after bilateral mastectomy for breast cancer, for which pancreaticoduodenectomy (PD) and lymphadenectomy were performed.

## Case report

**Fig. 1:**
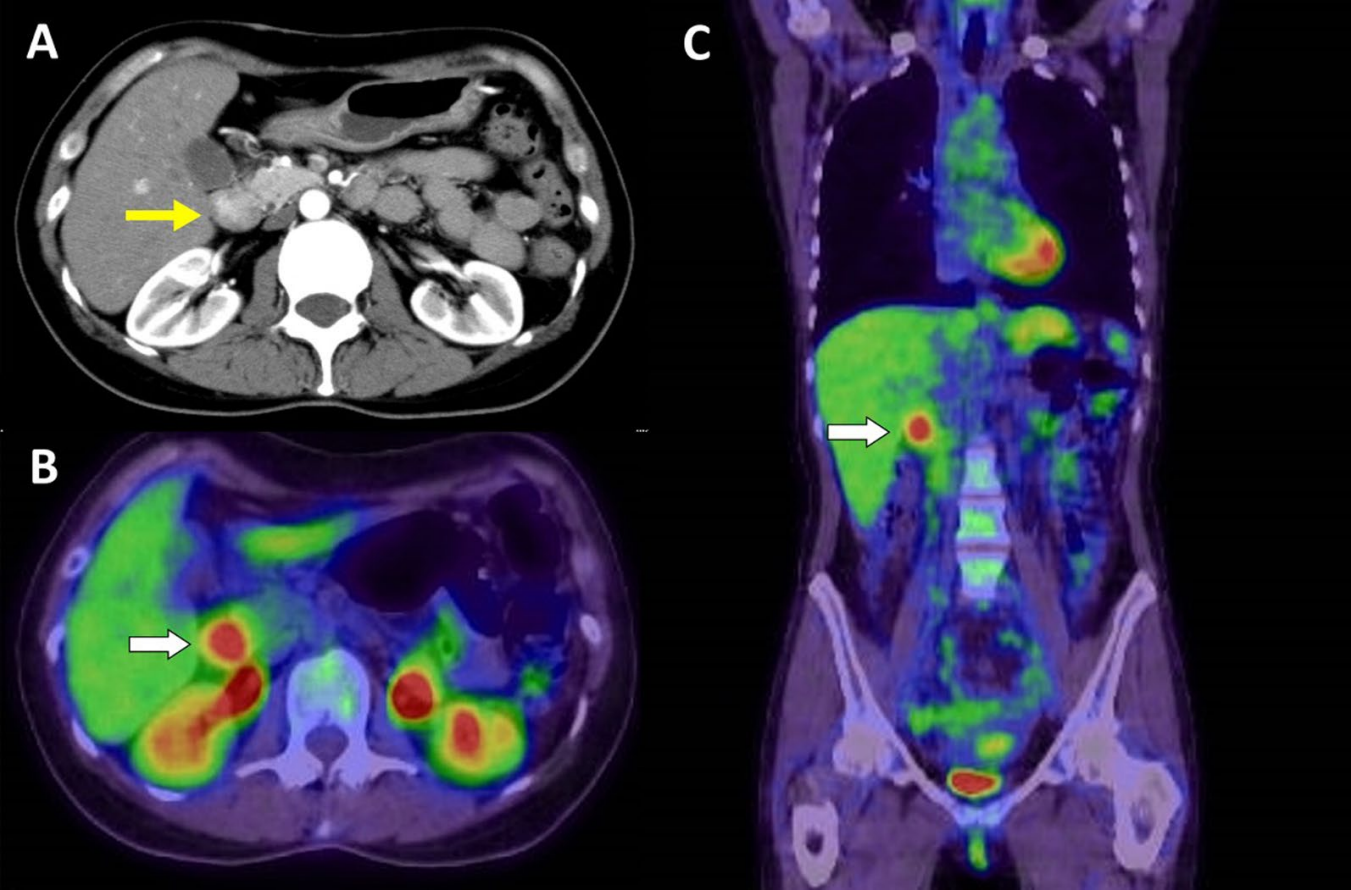
Computed tomography and 18-fluorodeoxyglucose–positron emission tomography images. **A** Contrast-enhanced computed tomography demonstrated a 20 × 20-mm duodenal mass (yellow arrow). **B**, **C** Demonstrated an accumulation of 18-fluorodeoxyglucose in this tumor with a maximum standardized uptake value of 5.78 (white arrows)

**Fig. 2:**
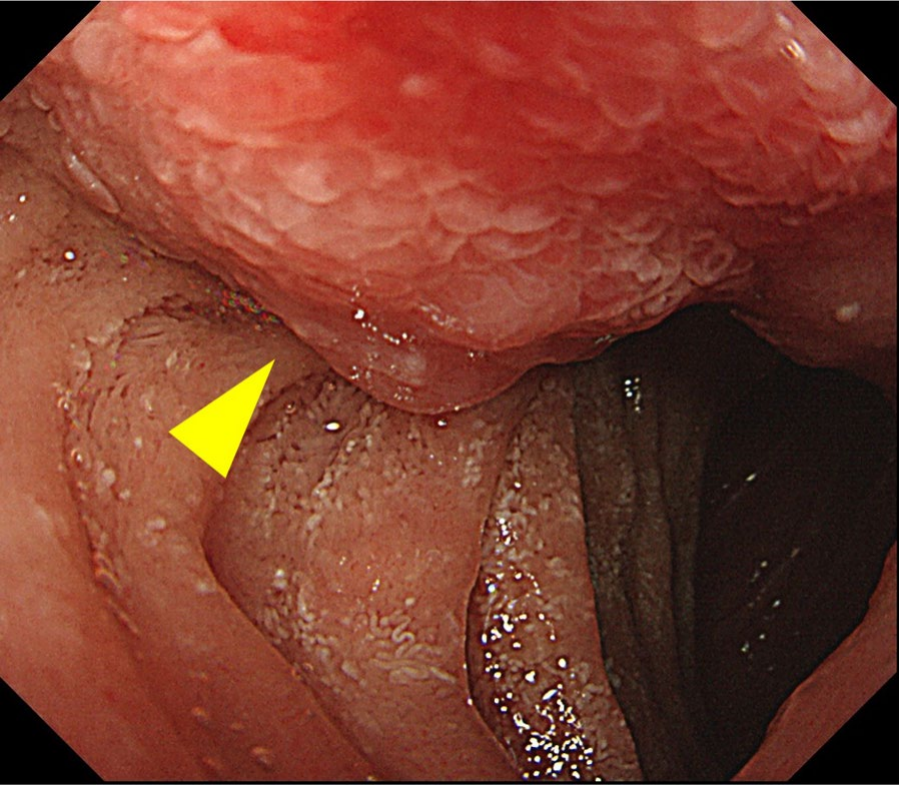
Endoscopic findings. Endoscopy revealed a 20-mm-diameter tumor in the second duodenal portion

**Fig. 3:**
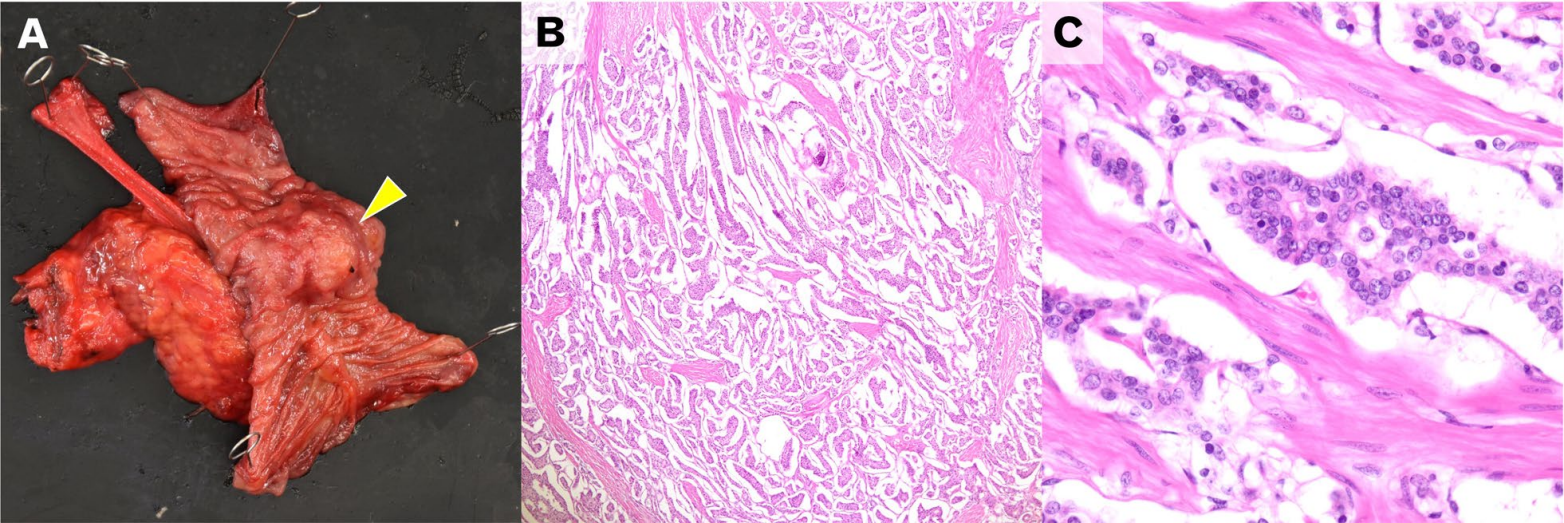
Gross finding of resected specimen and pathological findings. **A** Gross finding of neuroendocrine tumor (yellow arrowhead). **B** Pathological findings revealed displaying organoid pattern and lacking necrosis (HE stain, low-power field). **C** Pathological findings revealed composed of cells with minimal atypia in organoid pattern (HE stain, high-power field)

A 46-year-old woman was referred to us for examination and treatment of duodenal submucosal tumor (SMT). Her medical history was NF1, and her surgical history was bilateral mastectomy for breast cancer. She underwent a right total mastectomy and left partial mastectomy 9 and 5 years ago, respectively. Although her daughter was diagnosed with NF1, the medical history of her parents was not clear. After these operations, she was doing well without any recurrences. In addition, there were no signs of recurrence and clinical concerns, but she wanted to have systemic screening with 18-fluorodeoxyglucose–positron emission tomography (FDG–PET). FDG–PET showed FDG accumulation in the duodenal tumor unexpectedly. FDG accumulation had a maximum standardized uptake value of 5.78 (Fig. 1). These results suggest no evidence of metastasis. Endoscopy revealed a 20-mm-diameter tumor in the second duodenal portion (Fig. 2), and endoscopic biopsy suggested a NET G1. We performed PD and lymphadenectomy for complete resection (total operative time, 413 min; estimated blood loss, 155 mL), because preoperative evaluation suggested the potency of lymph node metastasis and invasion into the pancreas. Pathological findings suggested that a 22 × 17-mm NET had invaded the pancreas and lymph nodes (Fig. 3). The patient was discharged on postoperative day 8 without complications. She was doing well without recurrence and was followed up with PET tomography–computed tomography (CT).

## Discussion

We report a case of duodenal neuroendocrine tumor diagnosed during follow-up after bilateral mastectomy for breast cancer, for which PD and lymphadenectomy were performed. NF1 is an autosomal dominant disorder with a high risk of tumor formation. A high risk for breast cancer has already been established. However, the risk of abdominal tumors, such as GIST and NET, after mastectomy remains unclear. Furthermore, a follow-up strategy for other tumors after breast cancer has not yet been established. Thus, we have to consider two issues: what is the risk level of patients with NF1 and breast cancer for another de novo tumor, and how should we follow-up and treat patients with NF1 after mastectomy considering multiple neoplasms?

The risk of breast cancer in patients with NF1, particularly in young women, is very high [6, 7]. Johns Hopkins Hospital and some groups reported that patients with NF1 aged < 50 years have a high risk of breast cancer (standardized incidence ratio was 4.4–8.8, but the risk in patients aged > 50 years had no significant statistical difference) [6]. In addition, the proportional mortality rate of breast cancer patients with NF1 was 3.5 (95% confidence interval 1.3–7.7) [8]. In addition, another study suggested that 5-year overall survival of patients with breast cancer and NF1, after age and estrogen receptor expression level matched, was poorer than breast cancer patients without NF1 [9]. Furthermore, another study reviewed malignant neoplasms in women with NF1 and breast cancer [10]. In this study, 8 of 76 patients had various types of malignant neoplasms, and 5 patients had multiple neoplasms [10]. Only two patients with NF1 developed GI tumors: duodenal adenocarcinoma after 6 years of ductal carcinoma in situ of the breast and rectosigmoid adenocarcinoma after 5 years of invasive ductal carcinoma of the breast [10]. However, we could not find any other data on the risk of other GI tumors, including NETs, after mastectomy with NF1. In this case, the patient developed a neuroendocrine tumor 5 years after the last mastectomy.

A follow-up strategy for abdominal neoplasms in patients with NF1 and breast cancer has not been established. Sharif et al. recommended that screening with mammography for women from age 40 years should be considered, because women with NF1 aged < 50 years could be categorized with a moderately increased risk for breast cancer [11]. However, we need to consider other abdominal neoplasms, because some reports have revealed that GI tumors develop in 5–25% of patients with NF1 [3]. In particular, the reported incidence of symptomatic GI tumors, such as GIST and NET, is < 5% [3]. Thus, abdominal imaging, endoscopy, and endoscopic ultrasonography (EUS) should be considered for the follow-up of patients with NF1 after mastectomy; CT is not recommended for the usual follow-up of breast cancer [12, 13]. Daniel et al. reviewed 80 cases and reported tumor locations, types, and size; 78% of these tumors were > 5 cm, and common locations were the duodenum (60%) and ampulla of Vater (31%) [3]. Moreover, 26% of patients have multiple GI tumors [3]. Neuroendocrine tumors were only observed in 6% of patients, and most tumors were somatostatinomas (40%) and GIST (34%) [3]. Even if a < 2-cm SMT is not identified, it should be followed at 4–6 months with endoscopy or EUS [14, 15].

NET prognosis is dependent on derived locations and metastases [16, 17]. The prognosis of duodenal and periamupallry neuroendocrine tumors is not poor compared with that of NET originating from the pancreas and hind gut [16]. The Mount Sinai group analyzed population-based data (*n* = 3133) and revealed that the extent of lymph node involvement is a prognostic factor for gastroenteropancreatic NET [17]. Considering the risk of lymph node involvement in duodenal and periampullary neuroendocrine tumors, PPPD with lymphadenectomy is feasible for > 1-cm tumor and is recommended for tumors > 2-cm tumor [15, 18, 19]; tumors < 1 cm can be locally resected with endoscopy [15].

## Conclusions

Patients with a pathologic variant of NF1 should be followed carefully, because they are at a high risk of developing gastrointestinal tumors, including NETs, not only breast cancer. Patients with NF1 should be considered for GI screening and contrast-enhanced CT or MRI as a follow-up screening tool for over 5 years. Although endoscopy may be optimal in asymptomatic cases, endoscopic checkups should be considered in symptomatic cases. More cases should be accumulated to establish an effective follow-up for NF1 and to clarify the risk of other de novo tumors in patients with NF1.

## Data Availability

The data set supporting this article is available upon reasonable request from the corresponding authors.

## Abbreviations

CT: Computed tomography
EUS: Endoscopic ultrasonography
FDG–PET: 18-Fluorodeoxyglucose–positron emission tomography
GIST: Gastrointestinal stromal tumor
MRI: Magnetic resonance imaging
NET: Neuroendocrine tumor
NF1: Type 1 neurofibromatosis
PD: Pancreaticoduodenectomy
SMT: Submucosal tumor
